# A Prediction Model of the Incidence of Nonalcoholic Fatty Liver Disease With Visceral Fatty Obesity: A General Population-Based Study

**DOI:** 10.3389/fpubh.2022.895045

**Published:** 2022-06-23

**Authors:** Yang Zhou, Xiangping Chai, Tuo Guo, Yuting Pu, Mengping Zeng, Aifang Zhong, Guifang Yang, Jiajia Cai

**Affiliations:** ^1^Department of Emergency Medicine, Second Xiangya Hospital, Central South University, Changsha, China; ^2^Trauma Center, Second Xiangya Hospital, Central South University, Changsha, China; ^3^Outpatient Office, Second Xiangya Hospital, Central South University, Changsha, China

**Keywords:** nonalcoholic fatty liver disease (NAFLD), nomogram, obesity, prediction model, LASSO, risk score

## Abstract

**Objective:**

This study aimed to distinguish the risk variables of nonalcoholic fatty liver disease (NAFLD) and to construct a prediction model of NAFLD in visceral fat obesity in Japanese adults.

**Methods:**

This study is a historical cohort study that included 1,516 individuals with visceral obesity. All individuals were randomly divided into training group and validation group at 70% (*n* = 1,061) and 30% (*n* = 455), respectively. The LASSO method and multivariate regression analysis were performed for selecting risk factors in the training group. Then, overlapping features were selected to screen the effective and suitable risk variables for NAFLD with visceral fatty obesity, and a nomogram incorporating the selected risk factors in the training group was constructed. Then, we used the C-index, calibration plot, decision curve analysis, and cumulative hazard analysis to test the discrimination, calibration, and clinical meaning of the nomogram. At last, internal validation was used in the validation group.

**Results:**

We contract a nomogram and validated it using easily available and cost-effective parameters to predict the incidence of NAFLD in participants with visceral fatty obesity, including ALT, HbA1c, body weight, FPG, and TG. In training cohort, the area under the ROC was 0.863, with 95% CI: 0.84–0.885. In validation cohort, C-index was 0.887, with 95%CI: 0.857–0.888. The decision curve analysis showed that the model's prediction is more effective. Decision curve analysis of the training cohort and validation cohort showed that the predictive model was more effective in predicting the risk of NAFLD in Japanese patients with visceral fatty obesity. To help researchers and clinicians better use the nomogram, our online version can be accessed at https://xy2yyjzyxk.shinyapps.io/NAFLD/.

**Conclusions:**

Most patients with visceral fatty obesity have a risk of NALFD, but some will not develop into it. The presented nomogram can accurately identify these patients at high risk.

## Background

Recent epidemiological surveys estimated that the global prevalence of nonalcoholic fatty liver disease (NAFLD) is around 25%, with an expected increase to 33.5% by 2030 (1, 2). NAFLD is not only a major indication for liver transplant, but also leads to serious organ system injury outside the liver, such as endocrine system, kidney organs, cerebrovascular system, and musculoskeletal system (3–5). Several monotherapies and drug combinations are currently being evaluated for safety and efficacy in clinical trials targeting the pathogenesis of NAFLD (6). Since there are no Food and Drug Administration (FDA)-approved drugs, current treatment options focus on dietary restrictions, physical exercise, and lifestyle modification (6, 7). In the treatment and prevention of NAFLD, there is an urgent need for early detection and control of risk factors for NAFLD, and the prognosis of a predictive value of NAFLD-related risk factors needs to be further studied and explored. To date, NAFLD has become more common as the prevalence of obesity has increased (1, 8). Up to 80% of patients with NAFLD are obese, defined as body mass index (BMI) > 30 kg/m^2^. However, the distribution of adipose tissue plays a greater role in insulin resistance than the BMI. In morbidly obese (BMI > 40 kg/m^2^) individuals, large amounts of visceral adipose tissue (VAT) contribute to a high prevalence of NAFLD (9). Therefore, patients with NAFLD and visceral fatty obesity were in urgent need to screen the risk factors to prevent the incidence.

Numerous studies presented that obesity and dyslipidemia have been performed as the most effective risk factors for predicting NAFLD, including remnant cholesterol, triglyceride or high-density lipoprotein cholesterol, waist circumference, triglyceride glucose index, body mass index, waist-to-height ratio, and some other obesity and lipid-related indices (2, 10, 11). Some of the excess free fatty acids from the VAT tissue can be released into the portal venous system. Moreover, chronic low-grade inflammation and excess free fatty acids from VAT are considered to be the two most important factors leading to the progression of liver injury in NAFLD (12). In addition, lipid accumulation in the liver and VAT-secreted adipokines further promote inflammation and increase insulin resistance through the nuclear factor kappa B signaling pathway (13). Therefore, various pharmacological approaches using existing drugs (such as anti-obesity, anti-diabetes, cell protectants, and antioxidants) have been considered for the appropriate management of NAFLD in clinical practice (6).

Currently, the diagnosing and assessing the severity of NAFLD is liver biopsy, which is the gold standard; however, liver biopsy is not a routine procedure, and therefore, it is important that more practical screening techniques are developed. Recently, Zhou et al. established a nomogram to identify the earlier stage of liver fibrosis to avoid liver injury (14). Kim et al. build a nomogram to predict NAFLD in obese children (15). A nomogram is a graphical depiction of an individual predictive models, and although it has been developed for NAFLD in nonobesity adults or obese children, its application in the field of visceral fatty obesity is rare (15, 16). The objective of this research was to identify NAFLD early by simply noninvasive indicators and an online calculator to accurately detect earlier NAFLD in patients with visceral fatty obesity.

## Materials and Methods

### Study Population and Study Design

This study was a cross-sectional design according to the new research purpose. We used the NAGALA (NAFLD, longitudinal analysis) database to construct model predictions for nonalcoholic fatty liver risk in patients with visceral fat obesity. The original data were uploaded to the DRYAD database by Okamura et al. (17), who granted that the data could be analyzed freely based on the different scientific hypotheses. The purpose of this medical examination program, known as the Human Dock, was to identify chronic diseases and their risk variables and promote public health.

From 2004 to 2015, 15,744 individuals were enrolled in the original research and screened based on the inclusion and exclusion criteria. According to prior studies, this study enrolled 1,516 individuals in the NAGALA study after exclusion and explored the construction of noninvasive metrics-based predictive models of NAFLD in participants with visceral fat obesity. The exclusion criteria were as follows: (1) participants with alcoholic fatty liver disease, participates who drink more than 280 g per week (heavy alcohol consumption) and have fatty liver disease and viral hepatitis; (2) populations without visceral fat obesity: man, waist circumference <90; women, waist circumference <80 cm; (3) participates missing data; and (4) other exclusion criteria in original research (17).

The study was conducted in accordance with the Japanese Government's Ethical Guidelines for Medical and Health Research Involving Human Subjects and the Declaration of Helsinki. The study protocol was approved by the ethics committee of the Aichi Medical University School of Medicine.

### Exposure

In the original study and previously mentioned, the subjects' general clinical data were collected including age, gender, physical exercise behavior (habit of exercise), smoking status, body weight, fasting plasma glucose (FPG), systolic blood pressure (SBP), aspartate aminotransferase (AST), diastolic blood pressure (DBP), alanine aminotransferase (ALT), gamma-glutamyl transferase (GGT), high-density lipoprotein cholesterol (HDL-C), triglyceride (TG), hemoglobin A1c (HbA1c), and total cholesterol (TC). Smoking status was divided into three categories according to individuals' smoking history: never smoking, past smoking, and current smoking. Alcohol consumption was divided as four groups (18): more than 280 g per week was regarded as heavy alcohol consumption; 140–280 g per week as moderate; 40–140 g per week as light; and <40 g per week as no or minimal alcohol consumption.

### Outcome

In the original study, fatty liver was diagnosed by the findings of abdominal ultrasonography performed by trained technicians (17). NAFLD diagnosis was excluded drugs, viruses, or alcohol as the cause (10).

### Statistical Analysis

R software (version 4.1.0, http://www.r-project.org) and Stata 17.0 were used for all analyses. All enrolled populations were randomly divided into training groups and validation groups according to 70 and 30%, respectively. Continuous variables were shown as median (quartiles) or mean ± standard deviation, whereas categorical variables were shown as the percentage or frequency. The *t*-tests, chi-square tests, and Wilcoxon rank-sum tests were used based on the type of the variables.

A total of three steps were performed to build the nomogram. Step one: we used the univariate and multivariate logistic regression analyses for risk factors associated with fatty liver diseases in the training cohort. Step two: the least absolute shrink age and selection operator (LASSO) regression analyses were performed to identify risk variables by the shrinkage and variable selection for linear regression models in the training group. To achieve the subset of predictive variable, LASSO regression methods will exclude the irrelevant variables, which is estimated the value near zero. To avoid bias induced by the multivariate regression models, we only selected the overlapping features of the two models to develop the predictive nomogram and visualized them with a website version, which could provide clinicians with an intuitive and quantitative predictive tool to identify the patients at high risk of NAFLD. Finally, we validated the predictive efficiency and clinical ability of the nomogram in the internal dation cohort. Decision curve analysis (DCA) was used to evaluate the clinical utility of the nomogram prediction for training and validation groups, respectively. ROC curve, C-index, calibration curve, and DCA were used by bootstrap resampling to reduce overfitting bias. Statistical significance was set at *p* < 0.05.

## Results

### Participates' Characteristics

**Figure 1:**
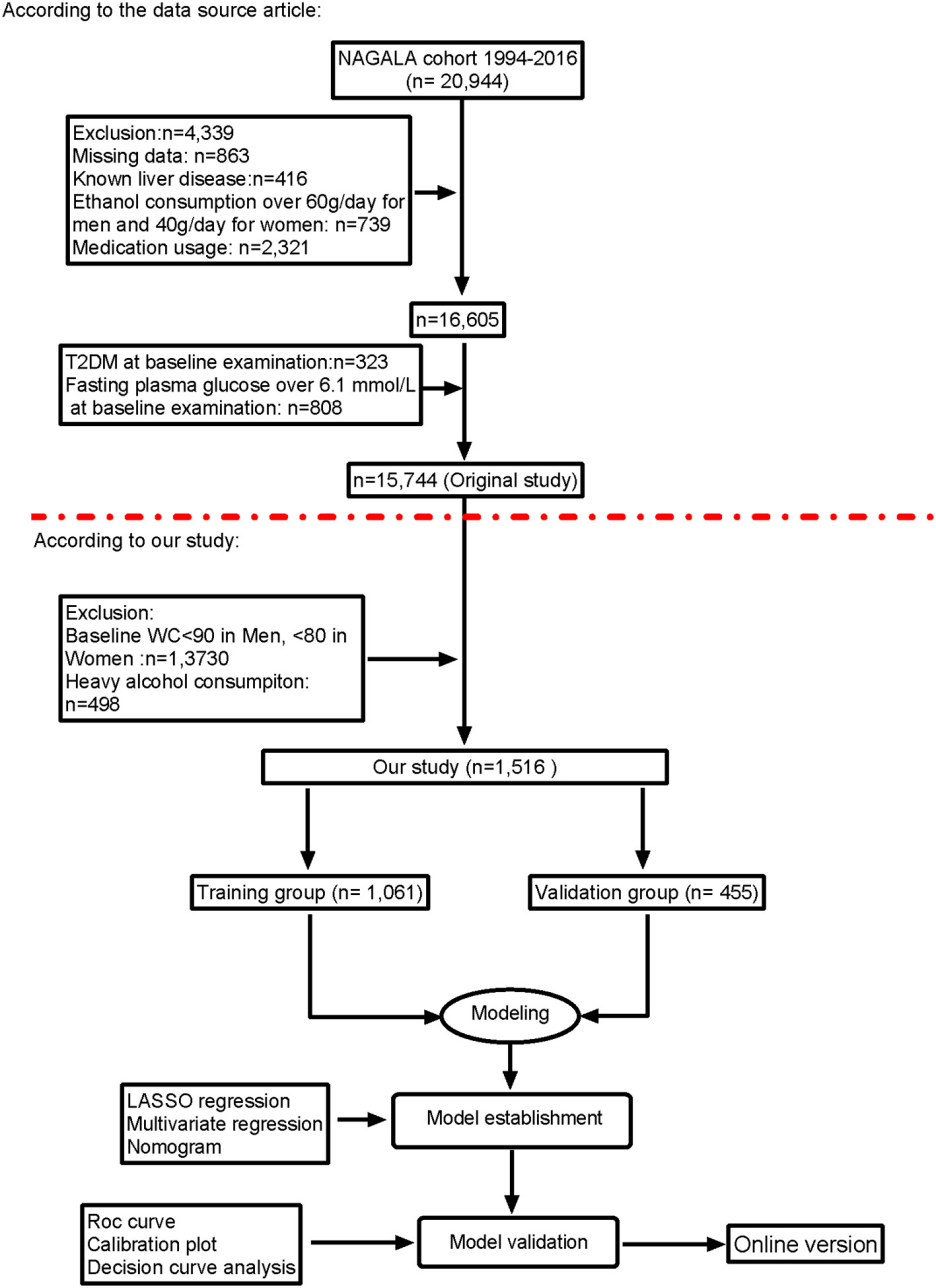
Flow diagram of study design.

The study included all of 1,516 participates with visceral fat obesity after exclusion and criteria (Figure 1), of which 1,061 participates were in the training group and 455 were in validation group. The mean age of the participates is 45.05 years, 62.16% of the eligible subjects were men, and 49.01% of the participates were with NAFLD. The incidence of NAFLD was 48.07 and 51.21%, respectively, of the training cohort and validation cohort. Baseline characteristics of the training and validation cohorts are summarized in Table 1.

**Table 1:** Demographic and clinical characteristics of study population in the derivation and validation cohorts.

**Characteristic**	**All**	**Training cohort**	**Validation cohort**	***P*-value**
No. of participants	1,516	1,061	455	
Age (years)	45.05 ± 8.73	44.61 ± 8.72	46.07 ± 8.67	0.003
Weight (kg)	72.01 ± 13.43	71.93 ± 13.41	72.21 ± 13.48	0.709
ALT (IU/L)	20.00 (14.00, 31.00)	20.00 (14.00, 31.00)	20.00 (15.00, 31.50)	0.989
AST (IU/L)	19.00 (15.00, 24.00)	18.00 (15.00, 23.00)	19.00 (15.50, 24.00)	0.474
GGT (IU/L)	16.00 (12.00, 25.00)	16.00 (12.00, 24.00)	17.00 (13.00, 26.00)	0.146
HDL (mg/dl)	50.49 ± 12.99	50.65 ± 13.10	50.13 ± 12.73	0.473
TC (mg/dl)	209.29 ± 33.71	208.02 ± 34.24	212.23 ± 32.27	0.026
TG (mg/dl)	85.00 (55.00, 127.00)	84.00 (54.00, 122.00)	86.00 (57.50,133.00)	0.192
HBA1C (%)	5.32 ± 0.33	5.32 ± 0.34	5.33 ± 0.33	0.679
FPG (mg/dl)	94.93 ± 7.21	94.86 ± 7.23	95.08 ± 7.18	0.588
SBP (mmHg)	122.10 ± 15.73	121.87 ± 15.97	122.65 ± 15.15	0.378
DBP (mmHg)	76.12 ± 10.99	75.97 ± 11.21	76.49 ± 10.46	0.399
**Gender [*****n*** **(%)]**				<0.001
Male	942 (62.16%)	487 (45.90%)	455 (100.00%)	
Female	574 (37.84%)	574 (54.10%)	0 (0.00%)	
**Nonalcoholic fatty liver disease**				0.262
No	773 (50.99%)	551 (51.93%)	222 (48.79%)	
Yes	743 (49.01%)	510 (48.07%)	233 (51.21%)	
**Habit of exercise, [*****n*** **(%)]**				0.015
No	1,348 (88.92%)	957 (90.20%)	391 (85.93%)	
Yes	168 (11.08%)	104 (9.80%)	64 (14.07%)	
**Smoking status, [*****n*** **(%)]**				0.048
Never	1,058 (69.79%)	759 (71.54%)	299 (65.71%)	
Past	218 (14.38%)	139 (13.10%)	79 (17.36%)	
Current	240 (15.83%)	163 (15.36%)	77 (16.92%)	

### Identification of Significant Features

**Figure 2:**
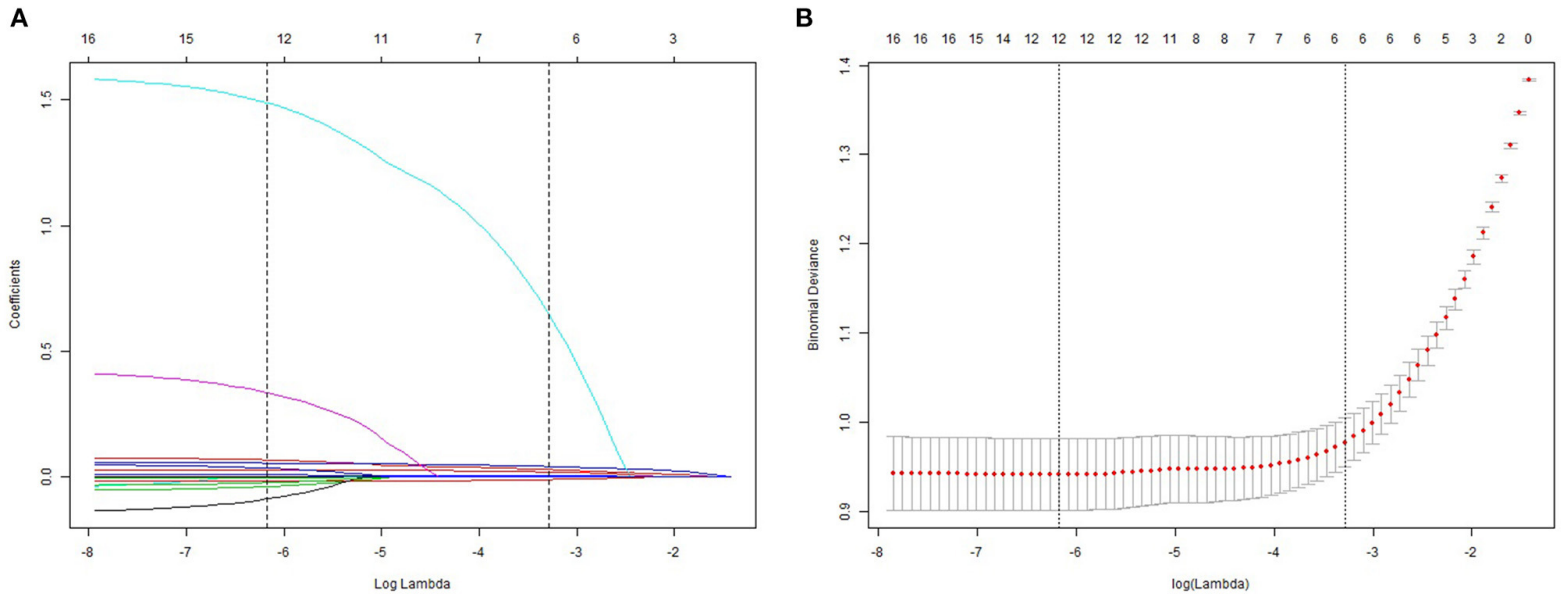
Demographic and clinical feature selection using the LASSO regression model. **(A)** 10-fold cross-validated error (first vertical line equals the minimum error, whereas the second vertical line shows the cross-validated error within 1 standard error of the minimum). **(B)** LASSO coefficient profiles of all the clinical features.

Univariate and multivariate logistic regression analyses were used in training cohort. The results suggested that weight, SBP, DBP, HbA1c, ALT, AST, and TG were dependently associated with NAFLD (Table 2). The LASSO regression was also performed by 10-fold cross-validation to select risk factors from basic characteristics in training cohort (Figures 2A,B). The factor is not near zero which was selected using the LASSO regression. A total of six factors were selected, including TG, HbA1c, FPG, HDL, ALT, and weight. The Lambda.lse is 0.0375 and the coefficients of the variables are shown in Supplementary Table S1.

**Table 2:** Univariate and multivariate logistic regression analysis for risk factors associated with fatty liver diseases in the training cohort.

**Characteristics**	**Univariable**	**Multivariable**
Age (years)	0.97 (0.96, 0.99) 0.0001	
**Gender [*****n*** **(%)]**		
Male	Ref	
Female	0.93 (0.73, 1.18) 0.5449	
Weight (kg)	1.10 (1.08, 1.11) <0.0001	1.06 (1.04, 1.08) <0.0001
SBP (mmHg)	1.03 (1.02, 1.04) <0.0001	0.97 (0.94, 0.99) 0.0046
DBP (mmHg)	1.06 (1.04, 1.07) <0.0001	1.05 (1.02, 1.09) 0.0041
**Habit of exercise, [*****n*** **(%)]**		
No	Ref	
Yes	0.96 (0.64, 1.44) 0.8378	
**Smoking status, [*****n*** **(%)]**		
Never	Ref	
Past	2.06 (1.43, 2.98) 0.0001	
Current	3.54 (2.45, 5.11) <0.0001	
HbA1c (%)	3.17 (2.17, 4.61) <0.0001	4.98 (2.86, 8.68) <0.0001
FPG (mg/dl)	1.10 (1.08, 1.12) <0.0001	
ALT (IU/L)	1.09 (1.08, 1.11) <0.0001	1.08 (1.06, 1.11) <0.0001
AST (IU/L)	1.11 (1.09, 1.13) <0.0001	0.95 (0.91, 0.98) 0.0018
GGT (IU/L)	1.07 (1.06, 1.09) <0.0001	
HDL (mg/dl)	0.94 (0.92, 0.95) <0.0001	
TC (mg/dl)	1.01 (1.00, 1.01) 0.0004	
TG (mg/dl)	1.02 (1.01, 1.02) <0.0001	1.01 (1.00, 1.01) <0.0001

### Developing and Validation of the Predictive Nomogram

**Figure 3:**
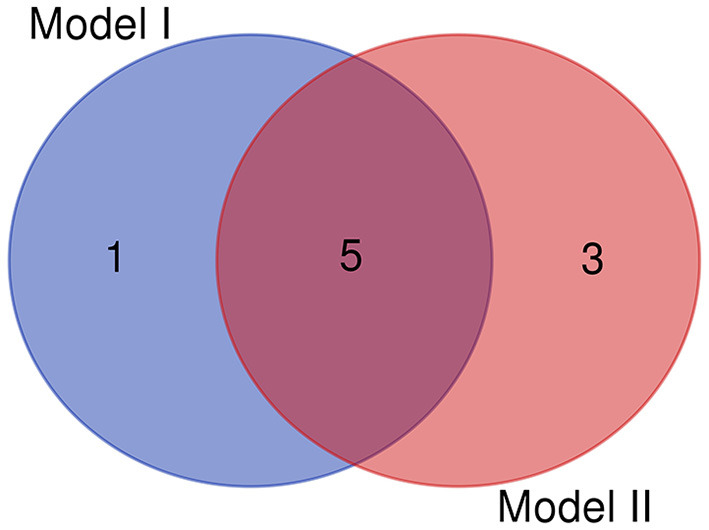
The overlapping features identified by the Model I (LASSO regression) and model II (multivariate logistic regression).

**Figure 4:**
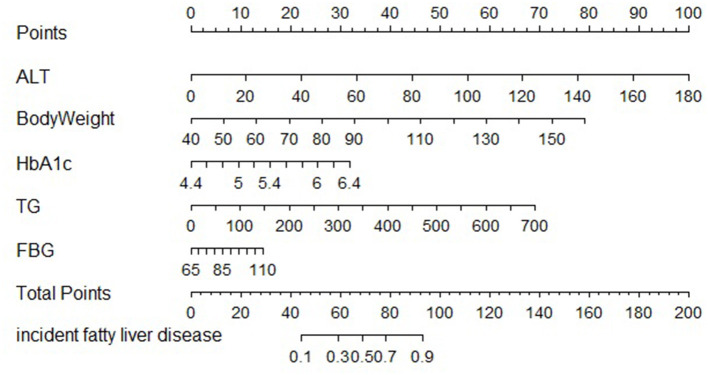
Nomogram for predicting the risk of NAFLD in adults with visceral fat obesity.

**Figure 5:**
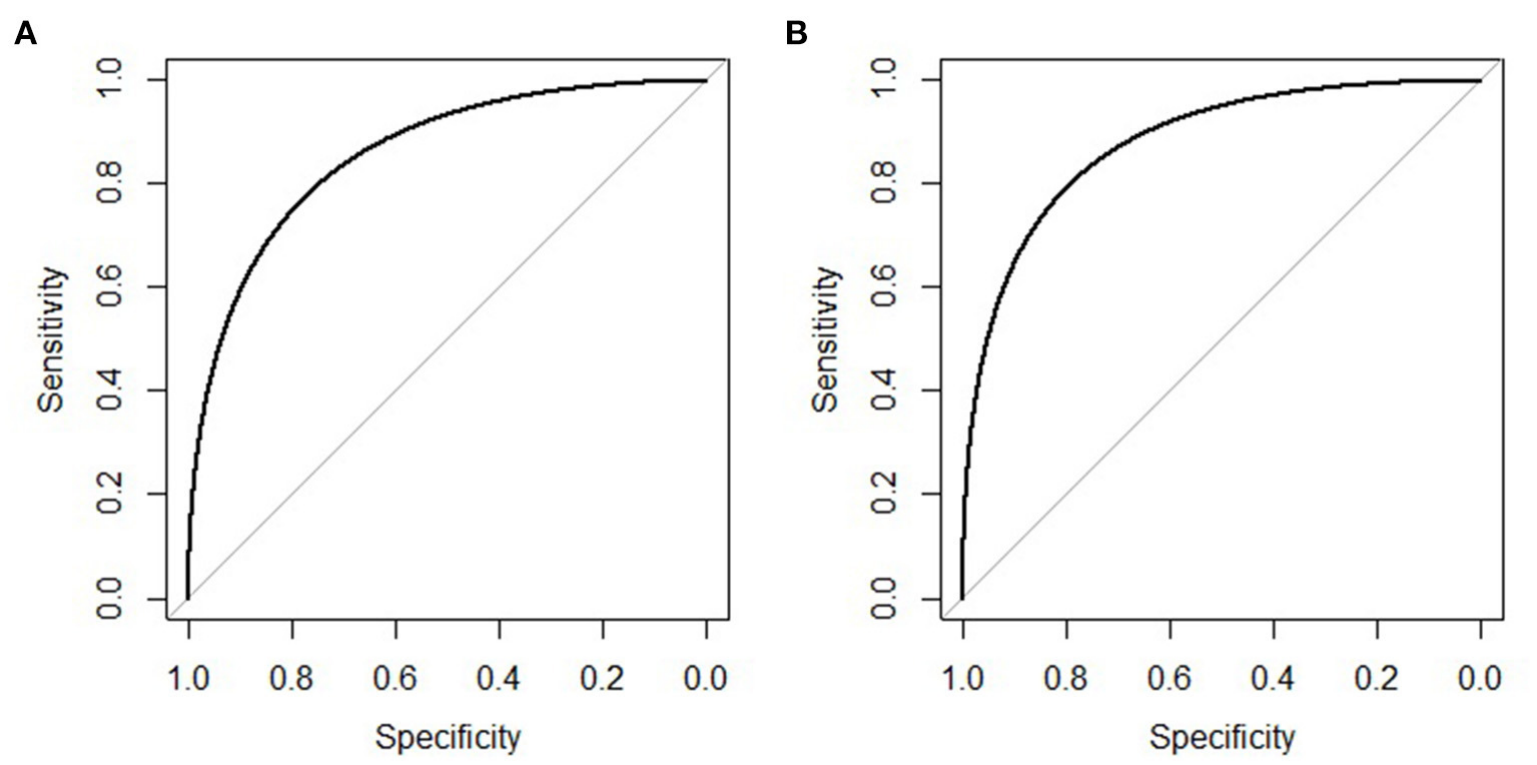
ROC curve of the nomogram in the training and validation cohort. **(A)** ROC curve of the nomogram in the training cohort. **(B)** ROC curve of the nomogram in the validation cohort.

**Figure 6:**
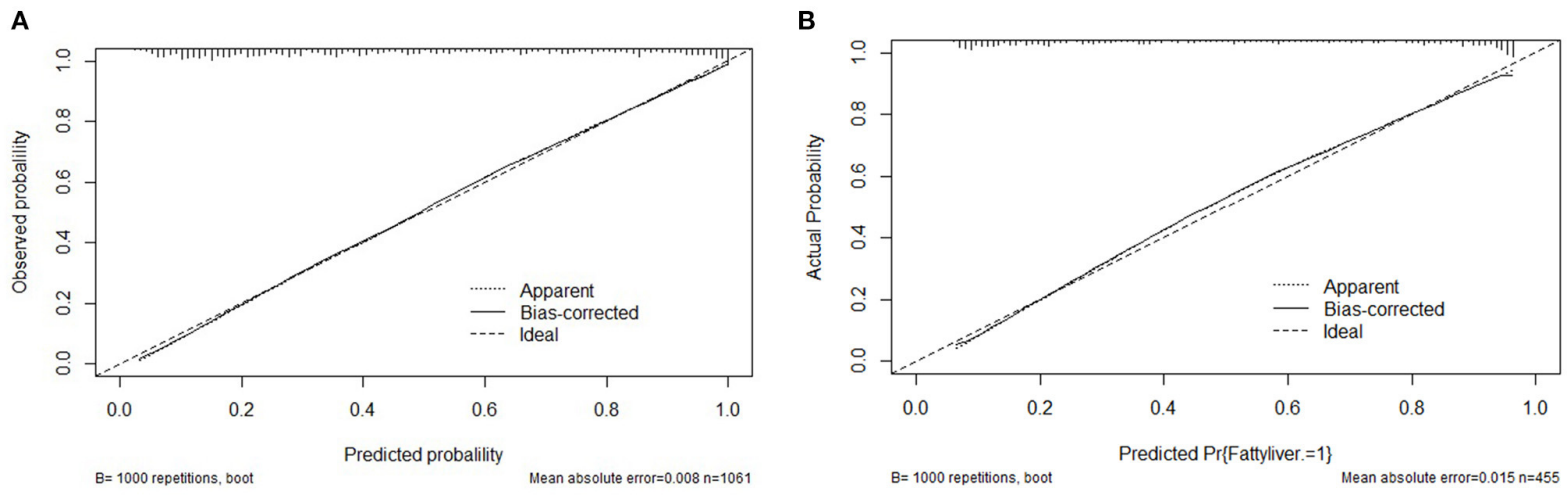
Calibration curves for the training and validation cohort models. **(A)** Calibration curve of the nomogram in the training cohort. **(B)** Calibration curve of the nomogram in the validation cohort.

**Figure 7:**
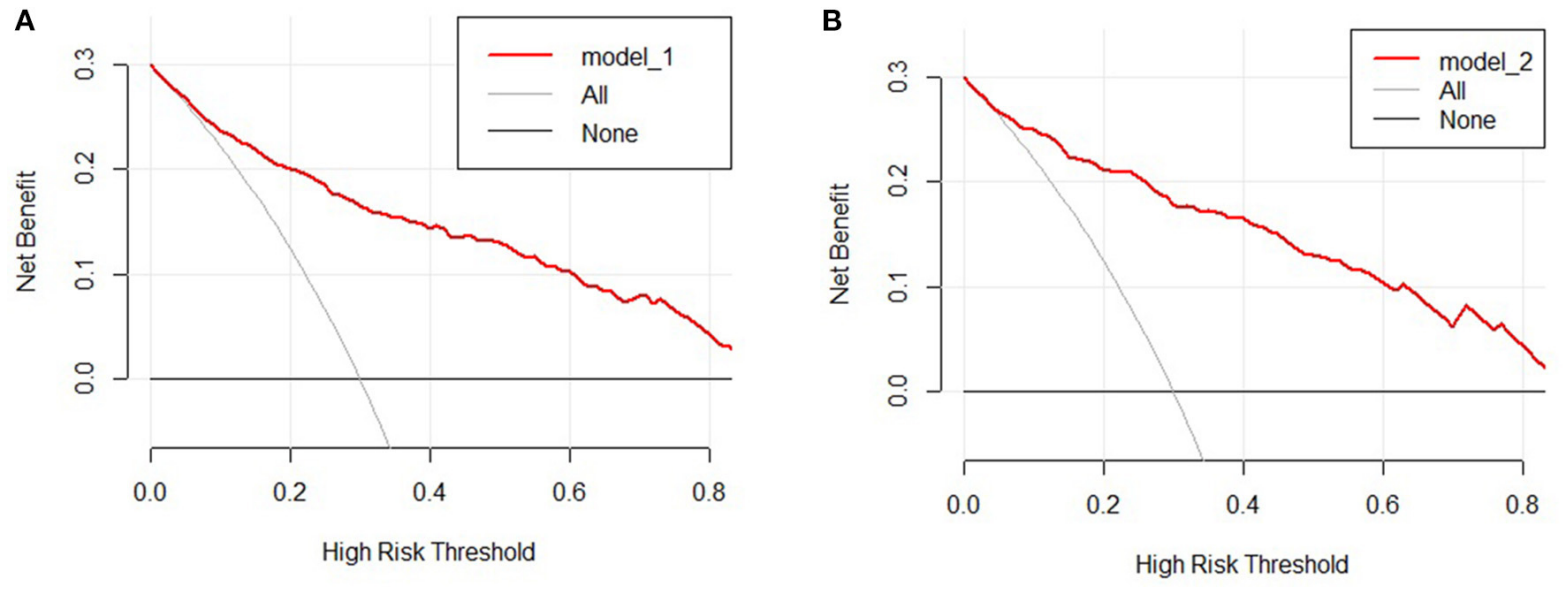
Decision curve analysis of the nomogram in the training **(A)** and validation cohorts **(B)**. The y-axis stands the net benefit.

The clinical features were selected from the overlapping section between multivariate model and LASSO regression (Figure 3). Based on the results of these two models, five risk factors were enrolled in the predictive nomogram for NAFLD (Figure 4), including HbA1c, FPG, TG, ALT, and bodyweight. C-index was applied to estimate the prediction model's performance. For training cohort, the area under the ROC was 0.863, with 95% CI: 0.841–0.885 (Figure 5A). In validation cohort, C-index was 0.887, with 95% CI: 0.857–0.888, and the result of bootstrap resampling validation confirmed that the nomogram's performance is stable in validation sets (Figure 5B). The calibration curves were obtained by constructing the calibration of the nomogram prediction model (Figures 6A,B). Decision curve analysis of the training (Figure 7A) and validation groups (Figure 7B) showed that the predictive model was more effective in predicting the risk of NAFLD in Japanese patients with visceral fatty obesity.

### Development of Webserver for Easy Access of Our New Model

To help the researchers and clinicians better use the nomogram, our online version can be accessed at https://xy2yyjzyxk.shinyapps.io/NAFLD/. By inputting clinical characteristics and reading the output data and tables generated by the web server, the predicted incidence of NAFLD in individuals with visceral fat can be easily determined.

## Discussion

This study developed a nomogram and an online version to predict the risk of NAFLD in visceral fatty obesity individuals. The nomogram consists of ALT, weight, HbA1c, TG, and FPG, which are related to obesity and metabolic-related indicators. To a certain extent, an internal cohort and calibration curves validated the clinical ability and predictive efficiency of the nomogram. In addition, decision curve analysis demonstrated that the predictive model was more effective in predicting the risk of NAFLD in Japanese patients with visceral fatty obesity.

Nomograms are widely used as a user-friendly decision-making tool in precision medicine. In a similar manner, Kim et al. used γ-glutamyl transpeptidase, homeostatic model assessment of insulin resistance, ALT, uric acid, and TG as the predictors built a nomogram that can be performed to detect for NAFLD in obese children (15). Earlier studies have shown that the presence of type 2 diabetes, obesity and age are associated with NAFLD and advanced fibrosis (1). Obese children in previous study were defined based on BMI; recently, relevant studies have indicated that BMI is an imperfect measure of obesity (19). Additionally, previous study found that waist circumference (WC) is more effective and strongly correlates with visceral adipose tissue (19), which were closely associated with NAFLD (12). Moreover, we used LASSO regression to identify risk variables, which would exclude the irrelevant variables (including variable selection and parameter elimination) and well suited for models showing high levels of multicollinearity, resulting in a more relevant and interpretable set of predictors (20). We only selected the overlapping features between LASSO and multivariate regression models to develop the predictive nomogram to avoid bias induced by the multivariate regression models. Overall, our study developed a nomogram that can be used to screen for NAFLD in adults with visceral fatty obesity using ALT, weight, HbA1c, TG, and FPG as predictors.

Alanine aminotransferase is one of the main liver function tests used to screen for hepatocellular damage and liver dysfunction (15, 21). Many studies have showed that ALT was an independent predictor of NAFLD (22). In addition, this research proved that ALT was a risk factor of NAFLD. ALT is a specific marker of hepatocyte damage and liver inflammation and is most closely associated with fat accumulation in liver (23). Therefore, in epidemiological studies, ALT is often used as an alternative marker for NAFLD (24). But a meta-analysis showed that 25% patients with NAFLD present as a normal ALT (25). Moreover, studies have shown that NAFLD patients with normal ALT levels also have histological features of disease progression (26, 27). Since most physicians assess liver risk for NAFLD based on ALT levels, NAFLD patients with normal ALT levels are easily neglected (25). Given these controversial reports and inconsistent conclusion about ALT levels in patients with NAFLD, we constructed the nomogram to predict the incidence of NAFLD among individual patients with visceral fat obesity.

Liver fat comes directly from the diet, adipose tissue lipolysis, or *de novo* lipogenesis. When triglyceride levels rise, free fatty acids in the blood also increase, which promotes fat decomposition. Hepatocyte damage characterized by NAFLD is driven by overload of major metabolic substrates (glucose and fatty acids) in the liver, leading to fatty acids entering pathways that promote cell damage and a dysfunctional response to pathways that lead to NAFLD in obese and diabetic patients (28). Previous study found that the plasma nonesterified fatty acid pool accounts for approximately 60% of the triacylglycerol content in the livers of patients with NAFLD (29). Although visceral adipose tissue is anatomically connected to the liver *via* the portal vein, most of nonesterified fatty acid in the portal vein originates in subcutaneous adipose tissue, even in subjects with visceral obesity (30–32). The increase in free fatty acids leads to a decrease in insulin sensitivity and histochemical stress, which in turn leads to an increase in insulin resistance in tissues (13). Body weight was considered as visceral fat and abdominal obesity have been associated with NAFLD. Recent accumulating evidence has indicated that body weight gain in overweight individuals increased the hyperlipidemia that induces NAFLD (33, 34). In our prediction model, body weight, FPG, HbA1c, and TG consisted of the NAFLD risk factor score. Therefore, the application of these risk predictors in our models is well founded.

The pathophysiology of NAFLD is multifactorial, and several mechanisms have been proposed. The main driver of NAFLD is overnutrition, which leads to the expansion of adipose depots and accumulation of heterotopic fat (6, 35). Lipid accumulation in hepatic steatosis or hepatocytes is the characteristic histological feature of NAFLD due to an imbalance between the inflow of fatty acids into the liver and their subsequent utilization (35). In this case, macrophage infiltration of visceral adipose tissue produces a proinflammatory state that promotes insulin resistance. In insulin resistance, improper lipolysis leads to increased delivery of fatty acids to the liver, which, together with increased deuterogenesis, overwhelms its metabolic capacity. An imbalance in lipid metabolism leads to the formation of lipotoxic lipids, which leads to inflammasome activation, cellular stress, tissue regeneration, apoptotic cell death, and subsequent stimulation of inflammation and fibrosis (36, 37). This complex disease involves an interplay between several factors; however, systemic insulin resistance is a major driver of hepatic steatosis in NAFLD. Thus, body weight, TG, FPG, and HbA1c were better enrolled in our predicted model, and C-index in validation cohort was 0.887.

The study has several strengths. In the first, it was the first study to build an accurate, personalized predictive model for NAFLD in patients with visceral fatty obesity in Japan. Then, our online version can be accessed at https://xy2yyjzyxk.shinyapps.io/NAFLD/, which can accurately identify these patients at high risk. Then, it was a large sample cohort and used multiple new methods to assess the performance of the nomogram. Also, there are some limitations in our study. First, this large-scale cohort study focused on a Japanese population. Whether the model exhibits better power in other ethnicities requires further validation in external cohorts. Second, our validation group consisted of the same individuals as the training group, which may suggest that the results were too optimistic. Third, due to the second analysis, some indicators, or novel biochemical markers, such as genetic factors, could not be adjusted in this study.

## Conclusion

We have established and validated a risk assessment system for characterizing the risk of NAFLD events, which has performed well. ALT, weight, HbA1c, TG, and FPG are in the top five for their projected contributions. We also constructed a predictive nomogram that provide an individualized risk assessment tool for NALFD in patients with visceral fatty obesity.

## Data Availability Statement

The original contributions presented in the study are included in the article/Supplementary Material, further inquiries can be directed to the corresponding author/s.

## Ethics Statement

As the authorization of the Ethics Committee of Murakami Memorial Hospital has been obtained in the previous study, this study does not need to be submitted for ethical approval again. Written informed consent from the participants' legal guardian/next of kin was not required to participate in this study in accordance with the national legislation and the institutional requirements.

## Author Contributions

JC and YZ: study concept and design and review and revision of the article. All authors: study conduct, data analysis, and writing of the first draft. All authors have read and approved the final manuscript.

## Funding

This work was supported by the Key Research and Development Program of Hunan Province (Nos. 2019SK2022 and 2020SK1014-2), Hunan Provincial Health Commission Project Fund (202115012883), Degree and Postgraduate Education Reform Project of Central South University (2021jy178), and Fundamental Research Funds for the Central Universities of Central South University (2020zzts291).

## Conflict of Interest

The authors declare that the research was conducted in the absence of any commercial or financial relationships that could be construed as a potential conflict of interest.

## Publisher's Note

All claims expressed in this article are solely those of the authors and do not necessarily represent those of their affiliated organizations, or those of the publisher, the editors and the reviewers. Any product that may be evaluated in this article, or claim that may be made by its manufacturer, is not guaranteed or endorsed by the publisher.
