# Supplementary material for: A Prediction Model of the Incidence of Nonalcoholic Fatty Liver Disease With Visceral Fatty Obesity: A General Population-Based Study
Source: Front Public Health. 2022 Jun 23;10:895045. doi: 10.3389/fpubh.2022.895045 (PMC9259946; doi:10.3389/fpubh.2022.895045)
Supplement: Supplementary file 1 [file Table_1.DOCX]

**Table S1** Coefficients and lambda.1se value of the LASSO regression based on the training cohort.

| Factors | Coefficients | Lambda.lse |
| --- | --- | --- |
| ALT (IU/L) | 0.0291574709886439 | 0.0375 |
| Weight (kg) | 0.041774246183374 |  |
| HDL(mg/dl) | -0.0104581179553062 |  |
| TG(mg/dl) | 0.00457609809522894 |  |
| HBA1C (%) | 0.647766785166129 |  |
| FPG (mg/dl) | 0.0194846963286171 |  |
